# The Prevalence of Scoliosis among Adolescent Chest Radiographs Obtained at Tikur Anbessa Specialized Hospital in 2019

**DOI:** 10.4314/ejhs.v33i4.10

**Published:** 2023-07

**Authors:** Mihretu Jegnie, Abay Mulu, Azmera Gissila, Misganaw Jegnie, Fetahi Minichil

**Affiliations:** 1 Department of Biomedical Sciences, College of Health Sciences, Debre Tabor University, Debre Tabor, Ethiopia; 2 Department of Anatomy, College of Health Sciences, Addis Ababa University, Addis Ababa, Ethiopia; 3 Department of Radiology, College of Health Sciences, Addis Ababa University, Addis Ababa, Ethiopia; 4 Department of Radiology, College of Medicine and Health Sciences, Hawassa University, Sidama, Ethiopia; 5 Department of Radiology, St. Peter's Specialized Hospital, Addis Ababa, Ethiopia

**Keywords:** adolescent scoliosis, spinal deformity, Ethiopia, Cobb angle, chest radiographs

## Abstract

**Background:**

Scoliosis is an abnormal side-to-side spinal curve of greater than or equal to 10^0^ Cobb angle. It is the most common spinal deformity in children and adolescents. Epidemiological evidence about scoliosis is scarce in Africa, including Ethiopia. This study was aimed at determining the prevalence of scoliosis among adolescents and analysing its association with age and sex using plain chest radiographs obtained for non-spinal reasons in Tikur Anbessa Specialized Hospital.

**Method:**

All non-tilted, non-rotated, and non-poorly penetrated digital plain chest radiographs of adolescents aged 10 to 19 years obtained at Tikur Anbessa Specialized Hospital between January 1 and December 31, 2019, were measured for the coronal Cobb angle. The data were cleaned, coded, and entered into SPSS version 26 for analysis. Chi-square, and linear regression, and logistic regression analyses were also carried out to evaluate the effect of sex and age on scoliosis.

**Results:**

The Cobb angles of 1,369 posteroanterior chest radiographs of adolescents were measured. Thirty (2.2%, 95% CI: 1.4%, 3.0%) of these were found to have scoliosis. The mean coronal Cobb angle was 2.27^0^±6.32^0^. There was no statistically significant difference between the prevalence of scoliosis in boys (2.21%) and girls (2.17%) (X^2^=0.003, P=0.954). Likewise, age did not show any statistically significant difference in the prevalence of scoliosis (X^2^=2.655, P=0.265).

**Conclusion:**

This study revealed that incidental finding of adolescent scoliosis in plain chest radiographs is common. Further study using whole spine radiography should be carried out to determine the true general population prevalence of scoliosis in Ethiopia.

## Introduction

Scoliosis is an abnormal side-to-side spinal curve of greater than or equal to 10^0^ Cobb angle in the coronal plane on a frontal erect radiograph. It is the most common spinal deformity seen in children and adolescents. The two major types of scoliosis are idiopathic and non-idiopathic. Idiopathic scoliosis is usually diagnosed by exclusion (1). It is the most common type (80%) and affects about 2–4% of the adolescent population (2). Non-idiopathic scoliosis is classified into congenital, neuromuscular, mesenchymal, and others (tuberculosis, ankylosing spondylitis, and degenerative scoliosis) (1,3). Based on its spinal location, scoliosis is classified as high thoracic, main thoracic, or thoraco-lumbar (4).

Globally, the prevalence of scoliosis in the general population ranges from 0.5% to 13% (5), whereas among school children, the prevalence ranges from 0.5% to 3% (6). Scoliosis affects approximately 2–4% of adolescents, with regional variations (7). Scoliosis affects approximately 6–9 million people in the United States (8). Studies showed that scoliosis affects 9.3% of adolescents in Chile (9), 10.4% in Turkey (10), 4.3% in Brazil (11), and 1.8% in Ethiopia (12). The overall prevalence of scoliosis in school children aged 10–14 years in Japan was 0.87% (13). In another prospective epidemiological study carried out in the USA, the incidence of adolescent idiopathic scoliosis (AIS) was found to be 4.5% (14). Evidence concerning the burden and determinant factors of scoliosis is scarce on the African continent.

Many studies have shown that females are more commonly affected by scoliosis than males (9,10,13,15–17). The overall ratio of girls to boys with scoliosis was 11:1 in Japan (13). A similar study in Turkey showed a significant difference in the prevalence of adolescent scoliosis between boys and girls (girls showing a higher prevalence) (10). Age has been found to be associated with an increased occurrence of scoliosis in adolescents. According to a study conducted in Sao Paulo, age has a significant influence on the development of scoliosis. Children aged 13–14 were 2.2 times more likely than those aged 10–12 to be diagnosed with scoliosis (17).

Radiography is the primary imaging modality employed for the diagnosis, monitoring, and management of scoliosis (18). The Cobb angle is the most commonly used and most accurate measurement of spinal curvature. It is obtained by measuring the maximal angle from the superior endplate of the superior end vertebra to the inferior endplate of the inferior end vertebra (2). Though Cobb is a widely used technique for the diagnosis of scoliosis using two-dimensional radiographs, it is highly affected by factors such as patient position, rotation, and radiographic technique (19). A total error of 2°–7° Cobb angle has been reported to result from variations in radiographic acquisitions and measurement error (2). The angle usually increases by an average of 5^0^ in the afternoon compared to its morning measurement. It has higher inter-observer (7.2^0^) and intra-observer (4.9^0^) variability if the end points cannot be defined (19).

The most frequently noted long-term sequelae of untreated AIS are curve progression, back pain, cardiopulmonary problems, and psychosocial concerns (20). An increased prevalence of back pain was observed in patients with a Cobb angle greater than 50° (21). According to a retrospective chart review study conducted in Montreal, Canada, back pain was a condition experienced by nearly half (47.3%) of the AIS patients (22). Untreated, severe progressive AIS could result in shortness of breath and devastating pain by restricting the area of the lungs due to severe spinal column and rib cage deformation (23). A higher risk of death has been observed among patients with thoracic curvatures greater than 100° by affecting lung function (21). Literature has also indicated the presence of an association between AIS, depression, anxiety, and neuroticism (24). Similarly, older untreated AIS patients are much less satisfied with their body image, have psychological distress, and have a poor quality of life compared to their controls (25).

Some developed countries in Europe, Asia, and America provide early scoliosis screening services to elementary school children in order to prevent the aforementioned long-term scoliosis sequelae. However, in developing countries, especially in Africa, there is no school based scoliosis screening program, and little is known about its burden. Thus, the main aim of this study is to determine the prevalence of scoliosis among adolescents and analyze its association with age and gender using plain chest radiographs obtained for non-spinal reasons in Tikur Anbesa Specialized Hospital. It is also aimed at determining the mean coronal Cobb angle in adolescents aged 10 to 19.

Epidemiological data on the prevalence of scoliosis among the Ethiopian population is scarce. The evidence generated from this study can be used as an input for policy implementation, program planning, and further study.

## Methods and Materials

**Study setting, period and design**: A retrospective cross-sectional study was conducted among digital plain chest radiographs of adolescents aged 10 to 19 in Tikur Anbesa Specialized Hospital (TASH) between 01 January 2019 and 31 December 2019. TASH is one of the largest and oldest public tertiary care hospitals in Ethiopia.

All posteroanterior (PA) chest radiographs of adolescents aged 10 to 19 obtained at the Radiology Department of Tikur Anbesa Specialized Hospital between January 1, 2019 and December 31, 2019 were included; while PA chest radiographs of patients with symptoms of back pain, spinal instrumentation, previous spinal surgery, or known pre-existing spinal disease were excluded. Radiographs obtained in the decubitus position were also excluded. Radiographs with poor image quality, such as rotated, tilted, or poorly penetrated films, were also excluded.

**Sample size determination and sampling technique**: All PA chest radiographs of adolescents aged 10 to 19 obtained at the Radiology Department of Tikur Anbesa Specialized Hospital between January 1, 2019

**Operational definitions**: and December 31, 2019 were assessed for scoliosis. About 1,421 adolescents had at least one plain chest radiograph in the year 2019. About 52 adolescents were excluded based on the aforementioned exclusion criteria.

**Variables of the study**: The dependent variables were Cobb angle and scoliosis, whereas age and sex were the independent variables.

**Figure 1:**
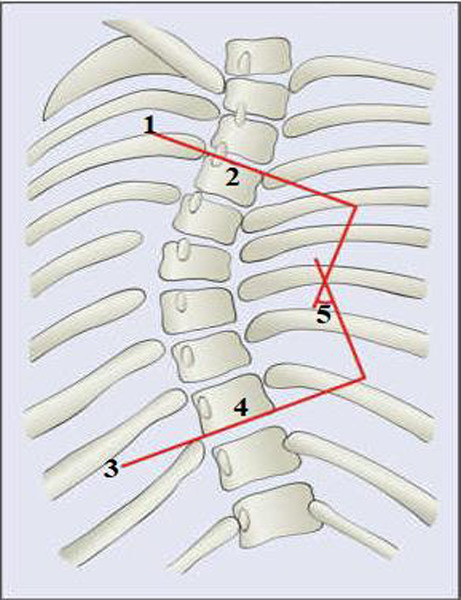
A schematic view of the spine showing how Cobb angle is measured (26) **Key**: 1: A line showing superior end plate of superior end vertebra; 2: superior end vertebra; 3: inferior end plate of inferior end vertebra; 4: inferior end vertebra; 5: Cobb angle

**Data collection procedure**: Digital Cobb angle measurements were obtained by using a software program at the workstation of the picture archiving and communication system (PACS) of Tikur Anbessa Specialized Hospital. As shown in Figure 1, the spinal curvature in the coronal plane was measured by plotting a line parallel to the superior endplate of the superior end vertebra and the inferior endplate of the inferior end vertebra if a curve is present, or between T1 and the lowest possible vertebral body visible if no obvious curve is observed. The curvatures were measured by two radiology residents. After two months, forty-eight radiographs were chosen at random to determine the inter-observer reliability of the measurement. The inter-observer reliability of Cobb angle measurement between the two raters was excellent (intra-class correlation coefficient = 0.88, 95% CI: 0.73-0.94).

**Scoliosis** is defined as the presence of a curvature measuring coronal Cobb angle equal to or greater than 10° irrespective of the aetiology. All forms of scoliosis (congenital, neuromuscular and idiopathic) were included in this study.

**Data processing and statistical analysis**: The data were cleaned, coded, and entered into SPSS version 26 for analysis. The mean Cobb angle was calculated. The overall prevalence of scoliosis was computed as a proportion of patients in terms of a percentage using the 95% confidence interval. The age- and sex-specific prevalence of scoliosis was also determined. A Pearson Chi-square test was performed to evaluate the association of scoliosis with sex and age. In addition, a linear regression analysis was performed between the Cobb angle measurement and the predictor variables age and gender. Logistic regression analysis was used to assess the association of scoliosis with age and gender. A p value of less than 0.05 was considered to be statistically significant. The data was presented using tables and graphs.

**Ethics approval and consent to participate**: The Department of Anatomy Research Ethics Review Committee (DRERC) granted ethical approval with reference letter DRERC/011/20. To obtain authorization for data collection, a formal letter was delivered to the Department of Radiology, College of Health Sciences.

## Results

Characteristics of study participants: About1, 369 PA plain chest radiographs obtained for adolescents aged 10-19 years old were assessed for scoliosis. The mean age was 14.56±2.96 years. About 41.2% of the radiographs evaluated were early adolescents (10 to 13 years). Based on sex, 723 (52.8%) were boys and 646 (47.2%) were girls.

Prevalence of scoliosis: The mean Cobb angle was 2.27±6.32^0^ ranging 0^0^ to 85.5^0^. About thirty (2.2%, 95% CI: 1.4%, 3.0%) of the adolescents were found to have scoliosis (curves equal to or greater than 10^0^). The prevalence of scoliosis in boys and girls were 2.21% (95% CI: 1.14%, 3.28%) and 2.17% (95% CI: 1.05%, 3.29%), respectively. The majority (76.7%) of the scoliosis cases were thoracic and the remaining (23.3%) were thoracolumbar. The prevalence of thoracic and thoracolumbar curves out of the 1, 369 chest radiographs were 1.7%, and 0.5% respectively. The scoliosis curves were classified based on severity as mild, moderate and severe as presented in Table 1.

**Table 1:** Scoliosis classification based on severity in Tikur Anbessa Specialized Hospital, 2022

Severity (Cobb angle)	Frequency			Percent	Female: Male ratio
	Female	Male	Total		
**Mild (10.00-20.00)**	6	4	10	33.3	1.5:1
**Moderate (20.01-45.00)**	6	5	11	36.7	1.2:1
**Sever (>45.00)**	2	7	9	30.0	0.29:1
**Total**	**14**	**16**	**30**	**100.0**	**0.875:1**

**Figure 2:**
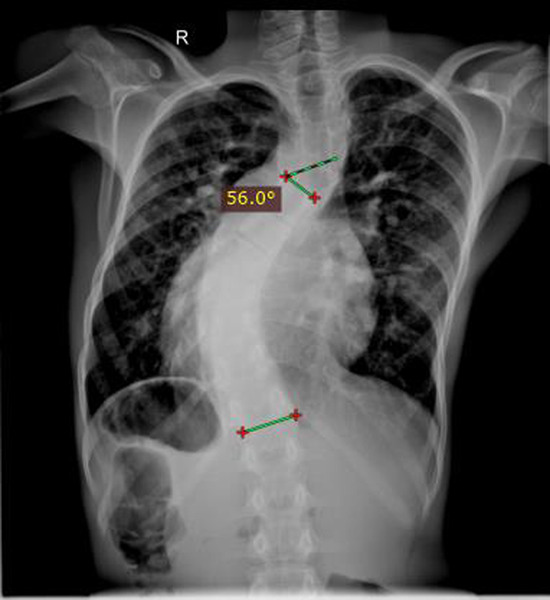
A plain chest radiograph of a 13-year-old boy with Cobb angle measurement of 56^0^

About 11 (36.7%) of the scoliosis cases were severe. About 11 (36.7%) of the scoliosis cases have additional minor curve. As it is presented in Figure 1 and Figure 2, the major spinal curves were classified as dextro (right convexity) and levo (left convexity). Majority of the major spinal curves (66.7%) were right convexity (dextro), while 10 (33.3%) of them were left convexity (levo).

**Association of scoliosis with sex and age**: There was no statistically significant difference between the prevalence of scoliosis in boys (2.21%) and girls (2.17%) (X^2^=0.003, p=0.954). Likewise, age category did not show any statistically significant difference in the prevalence of scoliosis (X^2^=2.655, p=0.265). Its prevalence, however; was higher in early adolescents (2.66%) and lower in mid-adolescents (1.12%) (Table 2).

**Table 2:** Association of scoliosis with gender and age in Tikur Anbessa Specialized Hospital, 2022

Variables	Category	Scoliosis (n, %)			Chi-square	p-value
		No	Yes	Total		
Gender	Male	707 (97.79)	16 (2.21)	723	0.003	0.954
	Female	632 (97.83)	14 (2.17)	646		
Age	10-13	549 (97.34)	15 (2.66)	564	2.656	0.265
	14-16	354 (98.88)	4 (1.120	358		
	17-19	436 (97.54)	11 (2.46)	447		
Total		**1339**	**30**	**1369**		

A linear regression analysis test was carried out to evaluate the effect of age and sex on coronal Cobb angle. Both age and sex did not influence coronal Cobb angle (age, p=0.585 sex, p=0.354) (Table 3). There was no statistically significant relationship between age and scoliosis as well as sex and scoliosis (Table 4).

**Table 3:** Bi-variable linear regression analysis to determine the relationship between age and sex with Cobb angle, among adolescent chest radiographs obtained at Tikur Anbessa Specialized Hospital, 2022

Variables	Crude OR (CI)	Sig.
Age	0.97 (-0.145, 0.082)	0.585
Sex	0.73 (-0.989, 0.354)	0.354

**Table 4:** Bi-variable binary logistic regression analysis to determine the relationship between age and sex with scoliosis, among adolescent chest radiographs obtained at Tikur Anbessa Specialized Hospital, 2022

Variables	Category	Scoliosis (n=1, 369)		Crude OR (CI)	Sig.
		Yes	No		
Age	10-13	549	15	1	
	14-16	354	4	.414 (0.136, 1.256)	0.119
	17-19	436	11	.923 (0.420, 2.031)	0.843
Sex	Male	707	16	1	
	Female	632	14	0.979 (0.474, 2.022)	0.954

## Discussion

This study was carried out by measuring the coronal Cobb angle of adolescent chest radiographs obtained at Tikur Anbessa Specialized Hospital to determine the prevalence of scoliosis among adolescents. The prevalence of scoliosis in adolescents was 2.2%. The mean Cobb angle among adolescent chest radiographs was 2.27^0^±6.32^0^. The majority (76.7%) of the scoliosis cases were thoracic. The majority (36.7%) of the scoliosis cases were severe. Age and sex did not have a statistically significant effect on the prevalence of scoliosis.

Thirty (2.2%) scoliosis cases were found. This finding is in line with a recent study conducted among elementary school children in Gondar that showed about 1.8% of the children aged 5 to 16 years old were positive for Adam's forward bend test (12). The lack of previous similar studies in Ethiopia limits the comparison of the findings of this study in the local context.

The present study showed a higher prevalence of scoliosis compared to the study done among schoolchildren aged 11–14 years in Tokyo, Japan (0.87%) (13). The prevalence of scoliosis in the current study is lower than that reported among schoolchildren aged 11–14 years in public schools in Brazil (4.3%) (11). Scoliosis was found in 10.4% of 1,045 plain PA chest radiographs of Turkish adolescents, which is higher than the current study (10). Similarly, the present study showed a lower prevalence of adolescent scoliosis compared to another study done among Chilean children aged 10-20 years old with a scoliosis prevalence of 9.3% (9). One possible reason for the lower prevalence of scoliosis in the current study could be the method used to identify it. A higher prevalence would be expected if Adam's forward test or scoliometer were used rather than radiographic diagnosis. Diagnosis of scoliosis using Adam's forward bend test and a scoliometer overestimates scoliosis compared to radiographic evaluations (16).

Another reason for the difference in the prevalence of scoliosis could be racial and geographic variation. Race/genetic factors were implicated in influencing scoliosis occurrence in various previous studies. For example, in a study by Kamtsiuris et al. (26), the prevalence of scoliosis among German children was higher (5.5%) than among immigrant children (3.5%). Similarly, Ratahi et al. (6) stated that AIS is more common in Europeans than in Polynesians. Scoliosis was seen more frequently in the Afro-American population (9.7%) than in the Caucasian population (8.1%) (27). Geographic and population-based differences in scoliosis prevalence rates have been noted in various literatures (28–30). Geographical variables such as temperature, humidity, and lighting influence human biology in the long run by expressing themselves in human cells through specific mediators (28). The prevalence of AIS rises in higher northern geographic latitudes and falls as one approaches the equator (29).

The mean coronal Cobb angle in the present study was 2.27^0^±6.32^0^ which is slightly lower than the study done in Turkey (4.4^0^) (10). The majority (76.7%) of the scoliosis cases were thoracic, which is consistent with the study done in Turkey (82.9%) (10), but slightly higher than the study done in Côte d'Ivoire (53.8%) (31). Almost all of the thoracic scoliosis cases (72 out of the 73) were thoracic in a study done in Chile (9), which is higher compared to the present study. Right convexity curves predominate (66.7%) in our study, which is similar to the finding of Yaokreh et al., 2022, in Côte d'Ivoire (65.1%) (31). Right-side curve predominance in our study can be explained by the fact that the pre-existing vertebral rotation pattern varies with age and occurs on the right side from adolescence onwards (32), which is consistent with the age group included in our study.

Scoliosis didn't show a statistically significant difference between girls and boys in this study, which is also supported by a previous study done in public schools in Goiânia, Brazil (11), and the municipality of Carlos Barbosa, Southern Brazil (16). However, a statistically significant difference in the prevalence of scoliosis was observed between girls and boys in other previous studies done in Chile (9), Turkey (10), Sao Paulo (Brazil) (17), and Japan (13). The proportion of females who had scoliosis was 13.9%, whereas only 5.3% of males had scoliosis (P< 0.01), with a female to male ratio of 3.2:1 in Chile (9). In a similar study done in Turkey, the prevalence of thoracic scoliosis was significantly higher in females than males (10). Scoliosis was detected in 2.15% of females and 0.47% of males, with a female-to-male ratio of 6.4:1 among 10–14-year-old public school students in three cities within the state of Sao Paulo, Brazil (17).

Several previous studies indicated that girls are more likely to have a severe grade of scoliosis than boys (14,33). On the contrary, severe grades of scoliosis were observed more in boys than in girls, with a male-to-female ratio of 3.5:1 in the present study. Because the minimum number of cases did not meet the chi-square test assumptions, a statistical test was not performed in our study to confirm the gender effect on the severity of scoliosis. Further study with larger samples of scoliosis cases is sought.

The age of the adolescents did not show statistically significant variation on the prevalence of scoliosis in the present study, which is in line with similar studies done in Ethiopia (12), Turkey (10), Chile (9), Japan (13), and Korea (33). However, age significantly influenced the development of scoliosis, according to a study done in Sao Paulo, Brazil (OR = 4.7, 95% CI: 1.8–12.2) (17).

Though the recommended approach to scoliosis diagnosis is primarily through physical examination and spine radiography, the inclusion of a large number of chest radiography samples in the present study will be a good proxy for the prevalence of scoliosis.

The strength of this study is its large sample size. As the study focused on archived radiographic images, it was not possible to order and obtain sideward-bending radiographs to evaluate whether the curves were structural or not. Since the study was conducted in a hospital among unhealthy adolescents who had undergone radiography, it must be clear that it could not represent the general healthy adolescent population.

In conclusion, this study revealed that the incidental finding of adolescent scoliosis on chest radiographs is fairly common. Radiologists shall routinely observe the spine for possible deformity and measure the Cobb angle for any plain chest radiograph. There was no statistically significant relationship found between scoliosis and age or gender. Further study using whole-spine radiography should be carried out to determine the true general population prevalence of scoliosis in Ethiopia.

## Figures and Tables

**Figure 3:**
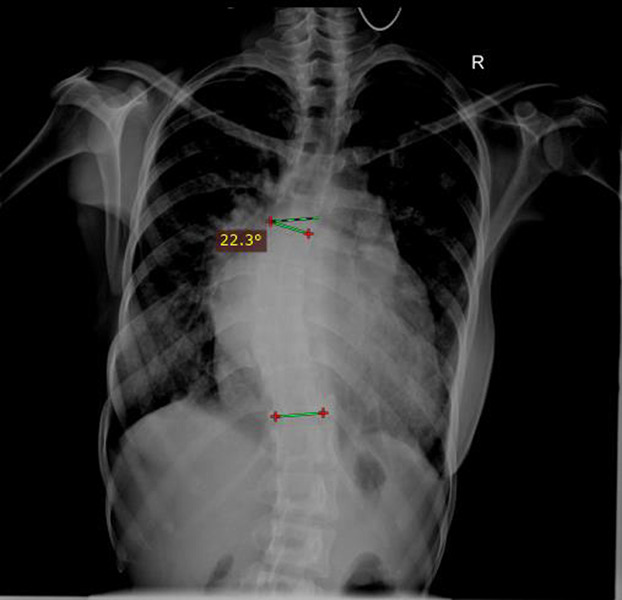
A plain chest radiograph of an 18-year-old girl with Cobb angle measurement of 22.3^0^
